# Ten years countdown to hepatitis C elimination in Belgium: a mathematical modeling approach

**DOI:** 10.1186/s12879-022-07378-3

**Published:** 2022-04-22

**Authors:** Dana Busschots, Erwin Ho, Sarah Blach, Frederik Nevens, Homie Razavi, Brieuc Van Damme, Thomas Vanwolleghem, Geert Robaeys

**Affiliations:** 1 Faculty of Medicine and Life Sciences, Hasselt University, Diepenbeek, Martelarenlaan 42, 3500 Hasselt, Belgium; 2 Department of Gastroenterology and Hepatology, Ziekenhuis Oost-Limburg, Genk, Belgium; 3 Department of Gastroenterology and Hepatology, Antwerp University Hospital, Antwerp, Belgium; 4 Center for Disease Analysis, Lafayette, CO USA; 5 Department of Gastroenterology and Hepatology, University Hospitals KU Leuven, Leuven, Belgium; 6 National Institute for Health and Disability Insurance (NIHDI), Brussels, Belgium; 7 Université Libre de Bruxelles (ULB), Brussels, Belgium; 8 Laboratory of Experimental Medicine and Pediatrics, University of Antwerp, Antwerp, Belgium

**Keywords:** Disease elimination, Health policy, Hepatitis C virus, Belgium

## Abstract

**Background:**

Chronic infection with the hepatitis C virus (HCV) remains a worldwide health problem. As a result, the World Health Organization (WHO) has set elimination targets by 2030. This study aims to examine the position of Belgium in meeting the WHO's targets by 2030.

**Methods:**

A Markov disease progression model, constructed in Microsoft Excel, was utilized to quantify the size of the HCV-infected population, by the liver disease stages, from 2015 to 2030. Two scenarios were developed to (1) forecast the disease burden in Belgium under the 2019 Base and (2) see what is needed to achieve the WHO targets.

**Results:**

It was estimated that the number of HCV RNA-positive individuals in Belgium in 2015 was 18,800. To achieve the WHO goals, Belgium needs to treat at least 1200 patients per year. This will only be feasible if the number of screening tests increases.

**Conclusions:**

Belgium is on target to reach the WHO targets by 2030 but will have to make sustained efforts. However, eradicating HCV requires policy changes to significantly increase prevention, screening, and treatment, alongside public health promotion, to raise awareness among high-risk populations and health care providers.

**Supplementary Information:**

The online version contains supplementary material available at 10.1186/s12879-022-07378-3.

## Introduction

Chronic infection with the hepatitis C virus (HCV) remains a worldwide health problem. As a result, the World Health Organization (WHO) has set elimination targets by 2030. These goals include reducing the number of new infections by 90% and the number of mortalities by 65% [1]. The estimated prevalence of HCV antibodies (Ab) and HCV-RNA in Belgium is 0.22% and 0.12%, respectively [2]. However, high-risk groups can continue to transmit the virus if risk behavior persists (e.g., people who inject drugs, PWID, and men who have sex with men) [3]. In addition, chronic HCV is often asymptomatic, and without treatment, liver damage can evolve into cirrhosis and potentially hepatocellular carcinoma (HCC) [4]. Vandijck et al*.* (2014) predicted that the economic impact of HCV and the disease at an advanced stage was expected to increase further. Costs related to HCV were forecast to peak at €126 million in 2026, whereas the costs of decompensated cirrhosis and HCC were predicted to increase until 2031 and 2034, respectively [5]. Timely interventions aimed at minimizing the health burden of an advanced disease could reduce these costs [5]. A Belgian 'Hepatitis C Plan' was developed in 2014 in response to the WHO's objectives. This plan aimed to (1) reduce transmission, (2) increase the number of HCV-positive patients aware of their diagnosis, and (3) enhance the patient care pathway and quality of life [6]. The majority of this plan's 23 predefined action points, including a screening policy and scientific data collection, have not yet been implemented. In fact, all efforts are still based on initiatives at the local level, and no national strategy is being implemented. A national, comprehensive HCV elimination plan could benefit from segmenting the population to treat, using concentrated elimination efforts within specific subgroups thereof. This concept is known as “micro-elimination”.

The introduction of interferon-free direct-acting antiviral agents (DAA's), with a high degree of sustained virologic response (> 95%), has facilitated the elimination of hepatitis C as a global epidemic [7]. Moreover, since January 2019, there are no longer any restrictions on the reimbursement criteria of DAA in Belgium. Recent modeling in Belgium showed that only people with hemophilia and transplant recipients were on track to achieve the WHO goals at the current rate of treatment [3]. The other groups added to the model (prisoners, people with HIV, migrants, hemodialysis patients and patients with advanced liver disease and PWID) would not reach the targets until 2056. They concluded that at least 8% of the population to be treated should receive treatment annually so that all subgroups would meet the WHO targets by 2030 [3]. However, these data were derived from unpublished data from national experts and a scientific literature review and thus provided only a limited epidemiological evaluation of the current Belgian situation. There have been continued efforts to collect more data on, e.g., HCV seroprevalence and treatment outcomes, owing to the increased attention to HCV in recent years. In recent years, studies have been conducted in both the general population and high-risk groups (e.g., migrants, prisoners, drug users, people living with HIV) [8–10]. These studies add new prevalence data combined with sociodemographic data. Moreover, we now also have the number of patients treated annually between 2015 and 2019. The availability of this data now allows us to perform more accurate modeling to determine Belgium's position in eliminating HCV. Is Belgium in a position to achieve the WHO objectives by 2030?

## Methods

### The Markov model and inputs

A Markov disease progression model, constructed in Microsoft Excel, was populated with data for Belgium and utilized to quantify the size of the HCV-infected population, by the liver disease stages, from 2015 to 2030. The model used has been described in detail previously [11, 12] with a brief summary included here. The model quantified the HCV infected population from 1950 through 2050 (outcomes summarized for the years 2015–2030), by liver disease stage and single year age and sex cohorts following the natural history of HCV (schematic overview available in Additional file 1). New infections entered the model annually as acute (incident) cases following an exposure to the HCV virus through a vertical (mother to child) or horizontal transmission source. After accounting for spontaneous clearance, chronic cases progressed through the model with annual outflows for death (all cause and liver-related) and patients achieving sustained virologic response (SVR) secondary to treatment. The model was populated and calibrated using Belgian-specific epidemiologic data gathered through the analysis of published and unpublished data and discussions with national experts. Two virtual meetings were conducted to review inputs, findings and analyses with the expert panel and incorporate their feedback. Historical incidence was determined based on known prevalence and backward calculations, while the future incidence was calculated in the model as a function of prevalence (see Additional file 1). Model parameters are described in Tables 1 and 2.

**Table 1:** The prevalence data of the different (risk) groups used in the model

Population	HCV RNA% (ref)	# Total estimated population in Belgium (ref)	# Infected Belgium
General population	0.12 [2]	11,405,000 [14]	13,686
IDU	25.00	10,000 [15]	2500
Prisoners	2.14 [10]	11,835 [16]	254
Migrants	0.17	15,204 [17]	26
PLHIV	6.27 [8]	18,908 [13]	1186
MSM with HIV	18.40 [8]	6301 [13]	1159
MSM	0.36 [13]	2924 [13]	10

*HCV* hepatitis C virus, *IDU* injecting drug users, *PLHIV* people living with HIV, *MSM* men who have sex with men

**Table 2:** HCV disease burden model input parameters

Category	Item	Source	Year	Base	Range*
Belgian Model Assumptions	Population by 5-year age and sex	[21]	1950–2050	–	–
	Mortality rate by 5-year age and sex	[21]	1950–2050	–	–
	Anti-HCV + prevalence	See Table 1 for base estimate (low [2]; high [22])	2015	0.3%	0.2–0.8%
	Viremic rate	[2]	2015	50%	–
	HCV prevalence by 5-year age and sex	[23] and WIV HepC Report	2004	Additional file 1 (pp 3)	–
	Annually treated	National reports (Sciensano)	2019	2459	–
	Total diagnosed	Input national experts	2015	43%	–
	Newly diagnosed (anti-HCV +)	[19]	2015	2278	–
Disease Burden Model Parameters (not country specific)	Standardized Mortality Ratio—Injection Drug Use	See Additional file 1 (pp3) [24–29]	1950–2050	10.0	9.5–29.9
	Standardized Mortality Ratio—Transfusion	See Additional file 1 (pp3) [30]	1950–2050	2.1	1.3–17.6
	Disease Progression—Acute to Spontaneous Clearance	See Additional file 1 (pp 6–7)	1950–2050	18.0%	15.0–45.0%
	Disease Progression—Mild to Moderate Fibrosis	See Additional file 1 (pp 6–7)	1950–2050	Varies by age	(−) 41% ( +) 53%
	Disease Progression—Moderate Fibrosis to Cirrhosis	See Additional file 1 (pp 6–7)	1950–2050	Varies by age	(−) 43% ( +) 90%
	Disease Progression—Cirrhosis to HCC	See Additional file 1 (pp 6–7)	1950–2050	Varies by age	(−) 26% ( +) 32%
	Disease Progression—Cirrhosis to Decompensated Cirrhosis	See Additional file 1 (pp 6–7)	1950–2050	Varies by age	(−) 30% ( +) 36%
	Disease Progression—Decompensated Cirrhosis to Liver Related Death	See Additional file 1 (pp 6–7)	1950–2050	Varies by age	(−) 20% ( +) 20%
	Disease Progression—HCC to Liver Related Death (year 1)	See Additional file 1 (pp 6–7)	1950–2050	70.7%	43.0–77.0%
	Disease Progression—HCC to Liver Related Death (subsequent years)	See Additional file 1 (pp 6–7)	1950–2050	16.2%	11.0–23.0%

*Ranges are only provided for parameters considered in the uncertainty analysis

#### Uncertainty analysis

Sensitivity analysis was conducted using Crystal Ball (version 11.1.3708.0), an Excel add-in by Oracle (see Additional file 2). β-PERT distributions were used for all uncertain inputs, including historical prevalence, standardized mortality ratios (SMR) and disease transition rates. The range around historical prevalence was selected based on published literature, with a wide range chosen to capture the uncertainty in published estimates. Ranges around disease burden model parameters (including SMR and disease transition rates) were identified through a previous literature search and chosen considering the natural history of disease. A Monte Carlo simulation with 1000 trials was used to estimate 95% uncertainty intervals (UIs).

#### HCV antibody prevalence and viremia prevalence

Between July 2013 and January 2015, residual sera were collected through clinical laboratories in a multiple disease seroprevalence study. A total of 3209 specimens collected by 28 laboratories were tested for HCV. In the Belgian general population HCV seropositivity was estimated to be 0.22% (95% CI: 0.09–0.54%), and prevalence of chronic HCV infection 0.12% (95% CI: 0.03–0.41) [2]. Data on chronic HCV infection were supplemented with estimates from various (unpublished) studies such as prisoners (2.14%) [10], intravenous drug users (25.00%), migrants (0.17%), general HIV population (6.27%) [8], HIV positive men who have sex with men (MSM, 18.40%) [8] and MSM on pre-exposure prophylaxis (0.36%) [13]. The Litzroth study was used in combination with data on high-risk groups to obtain an estimated prevalence for the country and was set by the experts at 0.33% for HCV Ab and 0.17% for HCV RNA. The absolute number of HCV infections in Belgium was 18,821 and was rounded down to 18,800, and was considered as starting prevalence in 2015 (Table 1). Transplantation data were also collected but did not change the forecasts or results.

#### Previously and annually diagnosed cases

Data on the percentage of previously (43%) and the number of newly diagnosed (2278) cases per year were provided by experts from the national (unpublished) surveillance data [18, 19].

#### Treated and cured patients

The national experts also provided the number of treated patients in Belgium by year from national unpublished data between 2015 and 2019, with 2459 patients treated in 2019. According to experts, treatment with direct-acting antivirals achieved a 98% sustained virologic response rate across all genotypes and disease stages [20].

### Scenario analyses

Two scenarios were developed to (1) forecast the disease burden in Belgium under the 2019 Base and (2) see what is needed to achieve the WHO targets.

**Fig. 1:**
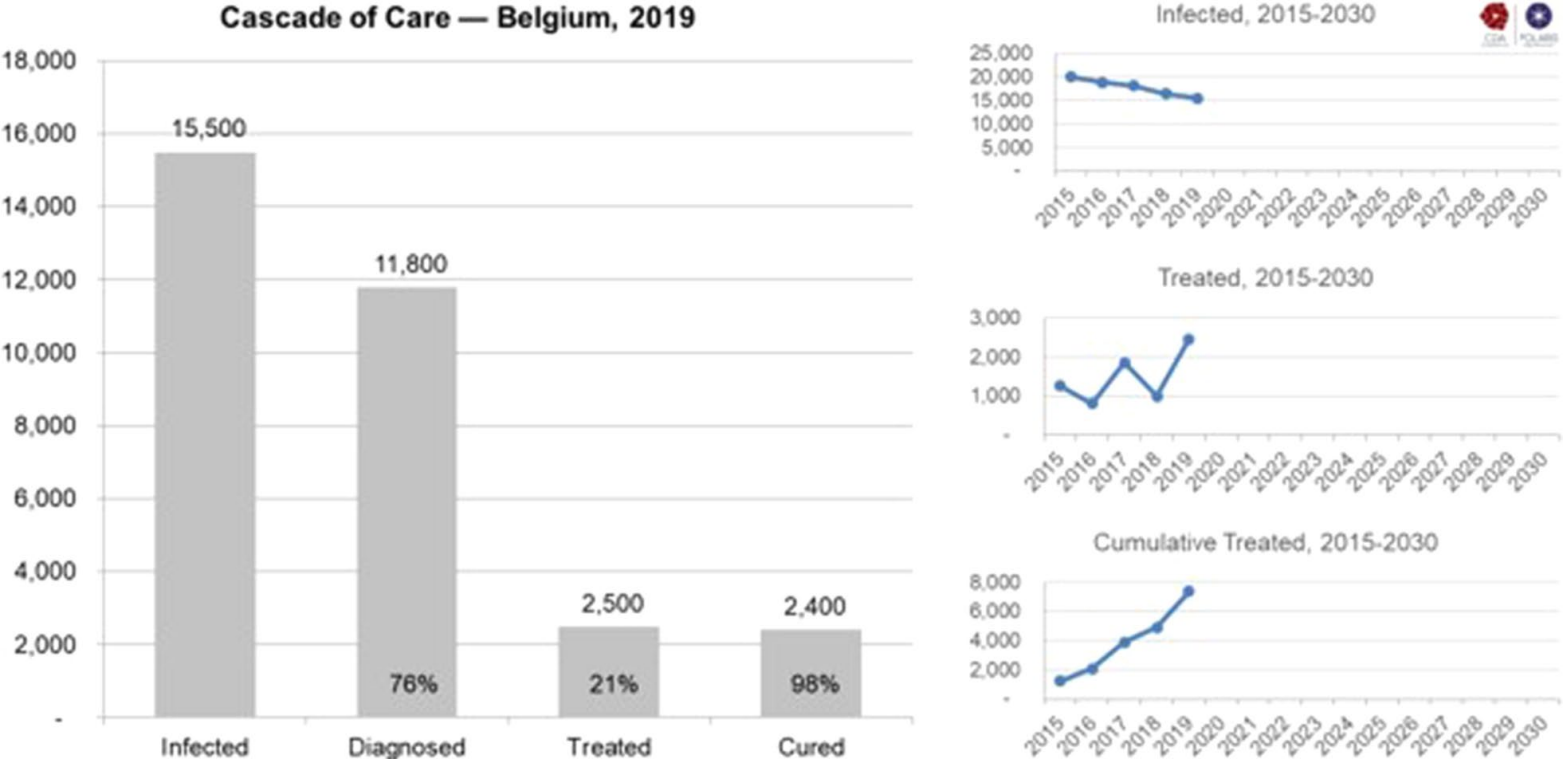
Cascade of care for hepatitis C in 2019 in Belgium

The HCV infection impact was calculated if there is no change to HCV treatment policies from 2015 to 2030. In 2015, patients staged ≥ F3 were eligible for treatment. In 2017, reimbursement criteria changed, and patients with confirmed stage F2 and patients with comorbidities such as HIV or hepatitis B co-infection, chronic kidney disease or extrahepatic manifestations were eligible. As of January 2019, treatment access was expanded to all infected patients in Belgium. These reimbursement adjustments are noticeable in the total number of patients treated each year between 2015 and 2019, with a significant increase from 2018 (∼989) to 2019 (∼2459) (Fig. 1). Nevertheless, a decline in treatments is expected if no additional efforts are made from 2020 onwards.

The number of newly diagnosed patients was calculated in the model, assuming a relatively consistent number of annual screening tests. Parameters for the 2019 Base scenario are summarized in Table 3.

**Table 3:** The number of people screened, diagnosed and treated in the 2019 Base

2019 Base	2015	2018	2019	2020	2021	2022–2030
Treated	1300	990	2500	1000	1000	930
Newly diagnosed	2300	1700	1300	1000	770	300
Screening tests	724,000	983,000	983,000	983,000	975,000	479,000

We modeled an intervention scenario to determine the extent to which the number of patients treated and diagnosed must increase from 2018 to meet WHO targets by 2030.

The number of screening tests that will be needed to meet the diagnosis target was calculated for each year. Genotype 1 accounted for 59.1% of all cases, followed by genotype 3, which accounted for 15.6%. In Belgium, however, treatment is done with pangenotypic DAAs.

The detailed description of the methodology can be found in Additional file 1.

## Results

The numbers of people who need to be screened, diagnosed, and treated under the WHO Targets scenario are shown in Table 4. The number of treated patients increased to a peak of 2,500 in 2019 after treatment access was expanded to all infected patients in Belgium.

**Table 4:** The number of people who need to be screened, diagnosed and treated to achieve the WHO Targets

WHO Targets	2015	2018	2019	2020–2025	2026–2030
Treated	1300	990	2500	1200	1200
Newly diagnosed	2300	1700	1300	800	80
Screening tests	724,000	983,000	983,000	784,000	2,495,000

*WHO* World Health Organization

Results are based on input from national experts (2015–2019) and modeling results (2020 onwards)

**Fig. 2:**
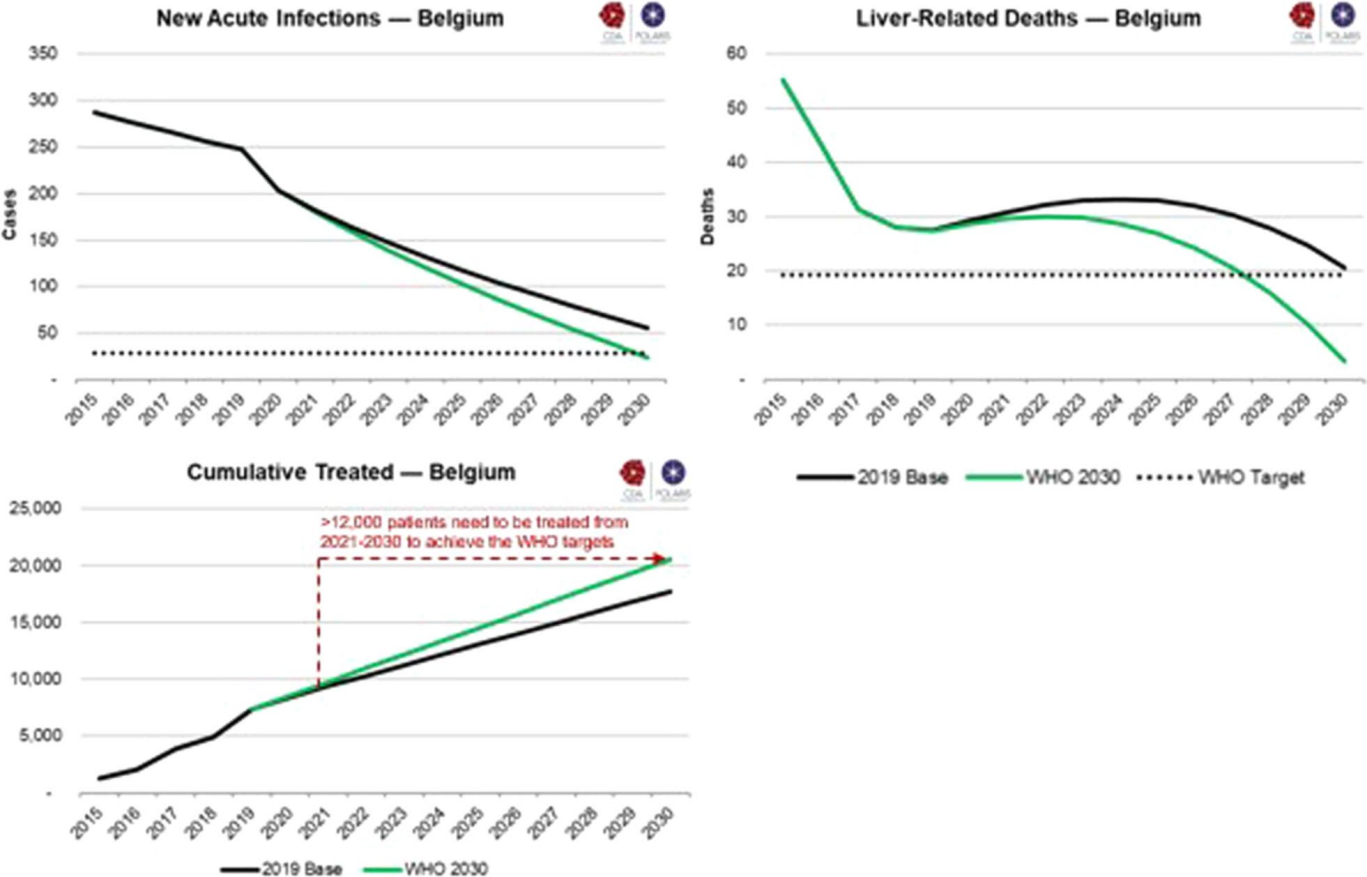
Graphs representing new infections, mortality and cumulative treatment rate for both scenarios

Considering a starting prevalence of 18,800 cases at the end of 2015, there would be an estimated 13,100 (95% UI: 7100–38,800) viremic cases at the beginning of 2020 (see Additional file 2). Belgium is close to achieving the WHO 2030 targets for incidence and mortality, but more efforts are needed (Fig. 2). To achieve the WHO goals, Belgium needs to treat at least 1200 patients per year. This will only be feasible if the number of screening tests increases. Thus, if we want to increase treatment rates, we need to increase annual screening. However, the number of people to screen will run out, explaining the drop in screening tests in Table 4. In addition, the yearly number of HCV-related deaths in Belgium is declining and estimated to be lower than 30 according to our model.

### Uncertainty analysis

The key drivers of uncertainty for model prevalence in 2020 were the input range around starting prevalence in 2015, and uncertainties around acute HCV to spontaneous clearance rate, and mild to moderate fibrosis transition rates (see Additional file 2). If prevalence in 2015 was at the high end (46,600) instead of the base value, then an average of 3600 people would need to be diagnosed with 90% (3240) initiated on treatment per year beginning in 2022 to achieve the WHO targets.

## Discussion

The mathematical model shows that Belgium is currently on track to meet the WHO targets by 2030, provided that at least 1200 patients continue to receive treatment each year. For this, a broad investment in screening and linkage to care remains a high priority.

An earlier publication showed that a doubling of the treatment rate was needed to achieve the targets [3]. However, this data was based purely on estimates. For this study, national experts provided the exact figures of treatment between 2015 and 2019. Moreover, in recent years studies have been conducted in both the general population and in high-risk groups (e.g., migrants, prisoners, drug users, people living with HIV), resulting in this study being more accurate [8–10].

Most patients diagnosed and registered at their hepatologist have already been treated, so potentially fewer patients will initiate treatment in 2020 than in 2019, even before COVID began impacting service delivery. Nevertheless, COVID will also have an impact on HCV care in Belgium. Due to strict COVID measures, screening programs were temporarily halted. In addition, regular care has been scaled down, causing a delay in treatment. However, recent research indicated that missed treatment would be more common in low-income countries, while most excess HCC and liver-related deaths would occur in high-income countries [31]. However, the focus remains on the WHO goals, so HCV care should be restarted and continued in Belgium whenever possible.

In 2014, a Belgian 'Hepatitis C Plan' was developed in response to the WHO's objectives [6]. Despite the endeavors, all efforts are based on local level initiatives and no national strategy is being implemented. Therefore, a new vision document was established in 2020. Based on an extensive literature review and multiple expert working groups, several policy recommendations were developed. The three main areas for which recommendations were developed are awareness, screening and treatment.

There is an urgent need in Belgium for broad-based information campaigns, e.g., posters in the general practitioner's waiting room and commercials on radio, TV, and social media. These campaigns should target the general public and primary care to raise awareness around HCV (transmission, risk, treatment) so that risk behaviors can be avoided and the virus detected more quickly. There is a need to continue working with patient organizations to put political pressure on the government.

Currently, screening appears to be the weakest link in HCV care in Belgium, especially in general practices (in Flanders). Since general practitioners have a pivotal role in prevention, diagnosis, and linkage to care, efforts to increase participation in HCV testing should also focus on general practitioners [32]. General screening of the entire population is not recommended because it is neither practical nor cost-effective, according to the Belgian Health Care Knowledge Centre (2014) [33]. Case findings will be essential to maintain the treatment rate and achieve the WHO targets. Thus, additional screening efforts should be made. Based on micro-elimination, risk groups should be targeted. The risk groups in Belgium were identified by Busschots et al. 2020 [3]. More specifically, the priority remains to focus on the groups with active high-risk behavior, such as PWID and MSM. Namely in these risk groups, there is a need for a regulatory framework that allows decentralized testing in a non-medical setting in primary care, for example, when social workers want to find out through a finger prick test whether a person is likely to be HCV seropositive. This is necessary to build a solid screening strategy in primary care and should be supported by mandatory registration of HCV and thus a national HCV registry. A national registry could map the progression to elimination as all initiatives are at a local level.

As of January 2019, access to treatment was extended to all infected patients in Belgium. The result was a significant increase in the number of patients treated in 2019. The removal of the reimbursement criteria and pangenotypic medication availability did ensure that nearly all patients previously diagnosed but were not eligible for treatment are now treated. Although treatment is practically on point, there is still a gap in the treatment of acute infections. Current treatment guidelines for acute cases allow for ongoing transmission before a patient is eligible for treatment (6 months post-infection). However, individuals are also infectious during those first 6 months and thus can cause ongoing transmission, especially in high-risk groups such as MSM and active PWID. Therefore, it is recommended that treatment eligibility is changed to three months post-infection [20]. Moreover, prisons are a unique environment to treat high-risk populations who may be otherwise hard to access [34]. This requires a coordinated universal test and treat program in the prison systems, currently absent in Belgian prisons. In this respect, Belgium could take an example from the Grand Duchy of Luxembourg, where care for infectious diseases is very well developed in prison [35].

This study has several limitations inherent in mathematical modeling. The inputs regarding epidemiology used in the model were not all published or available in the literature. A panel of experts was asked for input to overcome this limitation whenever the information was not available in peer-reviewed published literature. Secondly, we assumed that the future number of incident cases could be calculated in the model as a function of prevalence among patients with early stage disease. If HCV transmission patterns change drastically following the COVID-19 pandemic, this assumption may need to be revisited. In addition, a possible overlap between HCV Ab positives between the different studies was not considered. Finally, the model does not consider the possible impact on HCV disease burden, the progression of cured HCV patients, reinfection, comorbidities and extrahepatic manifestations. However, these limitations are common to similar studies and we believe that they do not pose a significant threat to the relevance and scope of the results presented.

## Conclusions

To conclude, Belgium is on target to reach the WHO targets by 2030 but will have to make sustained efforts. It incorporates policy changes to significantly increase prevention, screening and treatment of HCV, combined with a public health campaign to raise awareness among high-risk populations and health care providers. Screening should be scaled up using micro-elimination to reach specific groups. Implementation of a national HCV register to track progress and yearly screening, especially in groups with high-risk behavior, remains crucial.

## Supplementary Information

**Additional file 1**. Schematic overview of the Markov disease progression model.

**Additional file 2: Fig. S1.** Sensitivity analysis and 95% uncertainty intervals of Belgian viremic HCV prevalence.

## Data Availability

All data generated or analyzed during this study are included in this published article.

## Abbreviations

HCV: Hepatitis C virus
WHO: World Health Organization
Ab: Antibody
HCC: Hepatocellular carcinoma
DAA: Direct-acting antiviral
PWID: People who inject drugs
MSM: Men who have sex with men
