# Supplementary material for: Ten years countdown to hepatitis C elimination in Belgium: a mathematical modeling approach
Source: BMC Infect Dis. 2022 Apr 22;22:397. doi: 10.1186/s12879-022-07378-3 (PMC9026052; doi:10.1186/s12879-022-07378-3)
Supplement: Supplementary file 1 — Additional file 1. Schematic overview of the Markov disease progression model. [file 12879_2022_7378_MOESM1_ESM.docx]

Additional File 1

Ten years countdown to hepatitis C elimination in Belgium: a mathematical modeling approach - APPENDIX

*Adapted from: Polaris Observatory Collaborators. 2021. Global Change in Hepatitis C Virus Prevalence and Cascade of Care between 2015 and 2020: A Modeling Study Appendix. The Lancet Gastroenterology & Hepatology. [Accepted article in press]*

Contents

[Section 1. Forecasting Viremic HCV Prevalence 2](#_Toc93473381)

[References 11](#_Toc93473382)

# Section 1. Forecasting Viremic HCV Prevalence

**Indicator –** This analysis focused on estimating the viremic HCV infections, which reflects the presence of HCV RNA. The analysis used anti-HCV prevalence, serological evidence of past or present infection and the viremic rate in a Markov model to estimate viremic prevalence for the beginning of the year (1 January) 2020.

**Time period –** Building on the previous analysis, available data between 1 January 2015 and 31 March 2019 and input from national experts, were used in this analysis.

**Modeling HCV Prevalence –**A Markov model was used to forecast HCV prevalence over time. The prevalence of HCV is not constant over time. When incidence is higher than mortality and cured, the total number of infections will increase over time. The total number of infections will decrease over time when the opposite is true. The model was used to forecast the HCV prevalence at the beginning of 2020. The details of the model have been published previously (1-3).

**Required inputs –** The following inputs were required to build and calibrate a country model.

| Model input | Definition | Source |
| --- | --- | --- |
| Country/territory population by 5-year age cohort | The number of people in the country/territory, reported annually from 1950 to 2050 (by gender and 5-year age cohort) | UN Database |
| Mortality rate by  5-year age cohort | The percent of deaths among the total population, annually from 1950 to 2050 (by gender and 5-year age cohort) | UN Database |
| Anti-HCV + prevalence rate | Percent of the total population who are anti-HCV(+) | Published and unpublished data |
| Viremic rate | Percent of anti-HCV(+) individuals who are HCV RNA(+) | Litzroth 2019 (4) with data on high-risk groups (unpublished) |
| Age and gender distribution | HCV prevalence rate by age (5-year cohorts) and gender | De Maeght 2008 (5) and WIV HepC Report |
| Genotype distribution | Proportion of HCV RNA(+) population categorized by HCV genotype (out of 100%) | Published and unpublished estimates |
| Annually treated | Number of HCV infected individuals who have received treatment in a given year | National reports (Sciensano) |
| Total diagnosed | Viremic HCV cases diagnosed and alive in a given year | Input national experts |
| Newly diagnosed | Annual number of newly diagnosed HCV cases | KCE Report 173 (6) |
| Liver transplants | Annual number of liver transplants due to HCV | Input national experts; Eurotransplant Statistics Report Library |
| Hepatocellular Carcinoma (HCC) | Annual number of HCC incidence due to HCV | Belgian Cancer Registry adjusted for proportion attributed to HCV |

- **Prevalence by age –** Age distribution from De Maeght 2008(5) and sex distribution from the WIV HepC Report

De Maeght S, Henrion J, Bourgeois N, de Galocsy C, Langlet P, Michielsen P, Reynaert H, Robaeys G, Sprengers D, Orlent H, et al. A pilot observational survey of hepatitis C in Belgium. Acta Gastroenterol.Belg. 2008 Jan;71(1):4-8.Scientific Institute of Health WIV. Hepatitis C. 2010.

**Treated patients –** The national experts also provided the number of treated patients in Belgium by year from national unpublished data between 2015 and 2019, with 2,459 patients treated in 2019. According to experts, treatment with direct-acting antivirals achieved a 98% sustained virologic response rate across all genotypes and disease stages.[20]

**Liver transplants *–*** The annual number of liver transplants was gathered from national experts (unpublished) and adjusted for the percentage attributed to HCV. For examples of such an adjustment factor, see references listed here (7-12).

**Diagnosed patients –** Published and unpublished national data were used to identify diagnosed patients for HCV Ab and HCV RNA. However, no study was representative of the total Belgian population. For this reason, an expert panel was consulted to determine the number of diagnosed patients.

After the last year of available diagnosis data, forecasts for annual newly diagnosed infections were estimated as follows: The default assumption was that the number of screened patients (see screening module methodology) would remain constant. This resulted in a decreasing number of newly diagnosed infections over time.

**All-cause mortality –** The all-cause mortality rates by age and gender were gathered from the United Nations mortality database (13, 14). The rates were adjusted for an incremental increase in mortality due to intravenous drug use (IDU) and transfusion. A standard mortality ratio (SMR) of 10 (9.5-29.9) was used for the portion of the HCV-infected population who were active IDU between ages 15-44 (15-20). An SMR of 2.1 (1.3-17.6) was applied to all ages for the portion of the population infected due to transfusion (21). The number of people who inject drugs (PWID) and HCV prevalence among PWID was gathered through published studies (22-24) and divided by the total HCV infected population to estimate the percent of all HCV infections among active PWID.

**Markov model –** The Markov model described here has been used in collaboration with experts from more than 80 countries/territories to establish the burden of HCV at a national or regional level and the outcomes of these analyses have been extensively published. Additionally, the model has undergone independent review by modelers and epidemiologists in France, Greece, Australia, Egypt, Spain and Portugal and has been updated and modified according to their feedback. Following its inception in 2012 (25), the model underwent more than 80 revisions and updates before the release of the global prevalence estimates in 2016 (3). Since then, the model has undergone 10 additional revisions and updates to enhance its functionality and algorithms.

The Markov (disease progression) model was constructed in Microsoft Excel® (Microsoft Corp., Redmond, WA) to quantify the size of the HCV infected population, by the liver disease stages, from 1950-2050. The size and impact of the HCV infected population prior to 1950 were considered negligible for the purposes of this analysis. Microsoft Excel was selected as a platform due to its transparency, availability and minimal need for operator training. The disease progression was modeled using the flow shown in the figure below and calculations shown in Equation 1.

The model started with the annual number of acute infections that progressed to chronic HCV (viremic) infection after accounting for spontaneous clearance of the virus. The methodology to calculate incidence is described below. The progression of these new cases was followed along with all chronic infections from prior years. Unless specified, the scope of the model was limited to viremic, HCV ribonucleic acid (RNA) positive cases. Non-viremic cases (those exposed to the virus but spontaneously cleared the virus or were treated and cured) were not considered.

The number of new cases at each stage of disease (incidence) was calculated annually by multiplying the annual progression rates times the prevalent population (by age and gender) in the previous stage. Thus, the annual number of new F2 cases was calculated by multiplying the prevalent population in F1 (by age and gender) times the F1 to F2 progression rate (Equation 1). After one year, new cases were considered prevalent cases (after accounting for mortality and cured).

The prevalent population at each stage of disease was tracked by one-year age cohort and was allowed to age (progress to the next age cohort) each year. The progression rates were back-calculated using five-year age cohorts (as described below). In this model, the progression rate was assumed to be constant over the five-year cohort. Thus, for ages 5-9, the F1 to F2 progression rate was assumed to be constant.

The flow of the HCV disease progression model


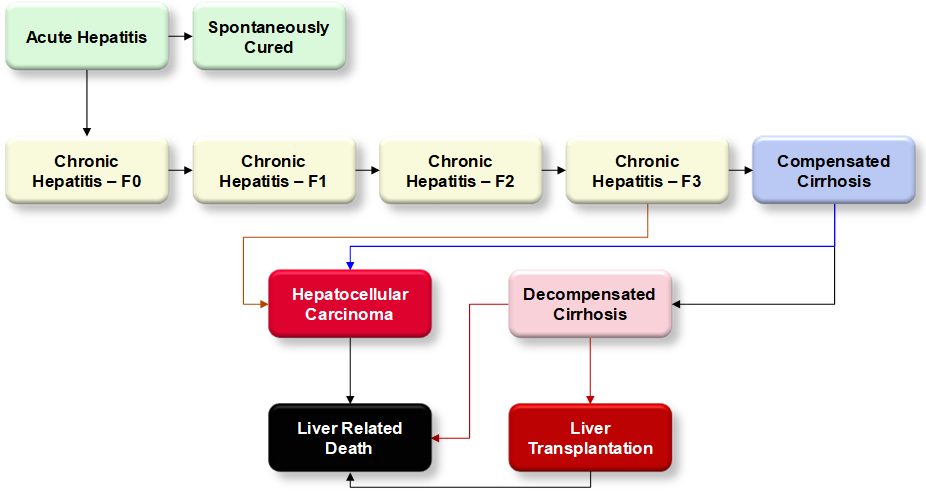


Equation 1. Annual prevalence (total cases) calculations by stage, year and age

${Total Cases}_{\mathrm{Stage}_{x} \mathrm{Year}_{y} {Age Cohort}_{z}}={Total Cases}_{\mathrm{Stage}_{x} \mathrm{Year}_{y-1} {Age Cohort}_{z-1}}+ {New Cases}_{\mathrm{Stage}_{x} \mathrm{Year}_{y} {Age Cohort}_{z}} -\mathrm{Cured}_{\mathrm{Stage}_{x} \mathrm{Year}_{y} {Age Cohort}_{z}}-{Background Mortality}_{\mathrm{Stage}_{x} \mathrm{Year}_{y} {Age Cohort}_{z}}-\mathrm{Progressed}_{\mathrm{Stage}_{x} \mathrm{Year}_{y} {Age Cohort}_{z}}-{Liver Related Mortality}_{\mathrm{Stage}_{x} \mathrm{Year}_{y} {Age Cohort}_{z}}$

where:

${New Cases}_{\mathrm{Stage}_{x} \mathrm{Year}_{y} {Age Cohort}_{z}}=\left( {Total Cases}_{\mathrm{Stage}_{x-1} \mathrm{Year}_{y-1} {Age Cohort}_{z-1}} \right)\left( {Progression Rate}_{\mathrm{Stage}_{x-1}\to\mathrm{Stage}_{x} {Age Cohort}_{z-1}} \right)$

$\mathrm{Cured}_{\mathrm{Stag}e_{x}\mathrm{Yea}r_{y}\mathrm{Ag}{e Cohort}_{z}}=\left( Total Cases_{\mathrm{Stag}e_{x}\mathrm{Yea}r_{y-1}\mathrm{Ag}{e Cohort}_{z-1}} \right)\left( {Age Eligibility Flag}_{\mathrm{Yea}r_{y-1}\mathrm{Ag}{e Cohort}_{z-1}} \right)\left( \frac{\mathrm{Cure}d_{\mathrm{Stag}e_{x}\mathrm{Yea}r_{y}}}{Total Age Eligible Cases_{\mathrm{Stag}e_{x}\mathrm{Yea}r_{y-1}}} \right)$

where:

$\mathrm{Cured}_{\mathrm{Stage}_{x} \mathrm{Year}_{y}}= \sum_{w=1}^{6} \left( {Total Treated}_{\mathrm{Genotype}_{w} \mathrm{Stage}_{x} \mathrm{Year}_{y}} \right)\left( \mathrm{SVR}_{\mathrm{Genotype}_{w} \mathrm{Year}_{y}} \right)$

${Background Mortality}_{\mathrm{Stage}_{x} \mathrm{Year}_{y} {Age Cohort}_{z}}= \left( {Total Cases}_{\mathrm{Stage}_{x} \mathrm{Year}_{y-1} {Age Cohort}_{z-1}}-\mathrm{Cured}_{\mathrm{Stag}e_{x}\mathrm{Yea}r_{y}\mathrm{Ag}e_{z}} \right)\left( Adjusted Background {Mortality Rate}_{{\mathrm{Yea}r_{y-1}Age Cohort}_{z-1}} \right)$

$\mathrm{Progressed}_{\mathrm{Stage}_{x} \mathrm{Year}_{y} {Age Cohort}_{z}}=\left( {Total Cases}_{\mathrm{Stage}_{x} \mathrm{Year}_{y-1} {Age Cohort}_{z-1}}-\mathrm{Cured}_{\mathrm{Stag}e_{x}\mathrm{Yea}r_{y}\mathrm{Ag}{e Cohort}_{z}}-{Background Mortality}_{\mathrm{Stage}_{x} \mathrm{Year}_{y} {Age Cohort}_{z}} \right)\left( {Progression Rate}_{\mathrm{Stage}_{x}\to\mathrm{Stage}_{x+1} {Age Cohort}_{z-1}} \right)$

${Liver Related Mortality}_{\mathrm{Stage}_{x} \mathrm{Year}_{y} {Age Cohort}_{z}}=\left( {Total Cases}_{\mathrm{Stage}_{x} \mathrm{Year}_{y-1} {Age Cohort}_{z-1}}-\mathrm{Cured}_{\mathrm{Stag}e_{x}\mathrm{Yea}r_{y}\mathrm{Ag}{e Cohort}_{z}}-{Background Mortality}_{\mathrm{Stage}_{x} \mathrm{Year}_{y} {Age Cohort}_{z}}-\mathrm{Progressed}_{\mathrm{Stage}_{x} \mathrm{Year}_{y} {Age Cohort}_{z}} \right)\left( Liver Related {Mortality Rate}_{{\mathrm{Yea}r_{y-1}Age Cohort}_{z-1}} \right)$

**Progression rates –** The progression rates by age, gender and fibrosis score were back calculated. Data from the UK were used for the percentage increase in progression rate by age and gender (26). However, this study only reported progression from chronic HCV to moderate chronic HCV and moderate chronic HCV to cirrhosis. These reported rates were modified using a meta-analysis of published work to calculate progression for F0, F1, F2, F3 and F4 (27). Finally, the modified progression rates were adjusted to fit historical HCC incidence by age and gender in the US (28) after adjusting for the portion of all HCC cases attributed to HCV (29).

The progression rates to end stage liver disease and liver-related deaths were based on previously published rates. Insufficient data were available to develop predictable rates by age and gender. Thus, the same rate was applied for all ages and genders (30-32). The table below lists all progression rates along with the uncertainty intervals.

HCV disease progression rates

| Back-Calculated Progression Rates – Males | | | | | | | | | | | | | | | | | | |
| --- | --- | --- | --- | --- | --- | --- | --- | --- | --- | --- | --- | --- | --- | --- | --- | --- | --- | --- |
| Age Cohorts | 0-  4 | 5-  9 | 10-  14 | 15-  19 | 20-  24 | 25-  29 | 30-  34 | 35-  39 | 40-  44 | 45-  49 | 50-  54 | 55-  59 | 60-  64 | 65-  69 | 70-  74 | 75-  79 | 80-  84 | 85+ |
| F0 to F1 | 5.3% | 5.3% | 5.3% | 5.3% | 5.3% | 5.3% | 5.3% | 5.3% | 13.9% | 13.9% | 17.1% | 17.1% | 19.4% | 19.4% | 21.8% | 21.8% | 21.8% | 21.8% |
| Low | 3.1% | 3.1% | 3.1% | 3.1% | 3.1% | 3.1% | 3.1% | 3.1% | 8.2% | 8.2% | 10.1% | 10.1% | 11.4% | 11.4% | 12.8% | 12.8% | 12.8% | 12.8% |
| High | 8.1% | 8.1% | 8.1% | 8.1% | 8.1% | 8.1% | 8.1% | 8,1% | 21.3% | 21.3% | 26.2% | 26.2% | 29.7% | 29.7% | 33.4% | 33.4% | 33.4% | 33.4% |
| F1 to F2 | 3.4% | 3.4% | 3.4% | 3.4% | 3.4% | 3.4% | 3.4% | 3.4% | 9.1% | 9.1% | 11.2% | 11.2% | 12.7% | 12.7% | 14.3% | 14.3% | 14.3% | 14.3% |
| Low | 2.0% | 2.0% | 2.0% | 2.0% | 2.0% | 2.0% | 2.0% | 2.0% | 5.3% | 5.3% | 6.6% | 6.6% | 7.5% | 7.5% | 8.4% | 8.4% | 8.4% | 8.4% |
| High | 5.3% | 5.3% | 5.3% | 5.3% | 5.3% | 5.3% | 5.3% | 5.3% | 13.9% | 13.9% | 17.1% | 17.1% | 19.4% | 19.4% | 21.8% | 21.8% | 21.8% | 21.8% |
| F2 to F3 | 5.4% | 5.4% | 5.4% | 5.4% | 5.4% | 5.4% | 5.4% | 5.4% | 14.3% | 14.3% | 17.5% | 17.5% | 19.9% | 19.9% | 22.4% | 22.4% | 22.4% | 22.4% |
| Low | 3.2% | 3.2% | 3.2% | 3.2% | 3.2% | 3.2% | 3.2% | 3.2% | 8.4% | 8.4% | 10.3% | 10.3% | 11.7% | 11.7% | 13.2% | 13.2% | 13.2% | 13.2% |
| High | 8.3% | 8.3% | 8.3% | 8.3% | 8.3% | 8.3% | 8.3% | 8.3% | 21.8% | 21.8% | 26.9% | 26.9% | 30.5% | 30.5% | 34.3% | 34.3% | 34.3% | 34.3% |
| F3 to C Cirrhosis | 5.7% | 5.7% | 5.7% | 5.7% | 5.7% | 5.7% | 5.7% | 5.7% | 9.3% | 9.3% | 9.3% | 9.3% | 10.4% | 10.4% | 20.0% | 20.0% | 20.0% | 20.0% |
| Low | 3.3% | 3.3% | 3.3% | 3.3% | 3.3% | 3.3% | 3.3% | 3.3% | 5.3% | 5.3% | 5.3% | 5.3% | 6.0% | 6.0% | 11.4% | 11.4% | 11.4% | 11.4% |
| High | 10.8% | 10.8% | 10.8% | 10.8% | 10.8% | 10.8% | 10.8% | 10.8% | 17.7% | 17.7% | 17.7% | 17.7% | 19.8% | 19.8% | 38.1% | 38.1% | 38.1% | 38.1% |
| F3 to HCC | 0.2% | 0.2% | 0.2% | 0.2% | 0.2% | 0.2% | 0.2% | 0.2% | 0.2% | 0.2% | 0.2% | 0.2% | 0.2% | 0.2% | 0.2% | 0.2% | 0.2% | 0.2% |
| Low | 0.2% | 0.2% | 0.2% | 0.2% | 0.2% | 0.2% | 0.2% | 0.2% | 0.2% | 0.2% | 0.2% | 0.2% | 0.2% | 0.2% | 0.2% | 0.2% | 0.2% | 0.2% |
| High | 0.3% | 0.3% | 0.3% | 0.3% | 0.3% | 0.3% | 0.3% | 0.3% | 0.3% | 0.3% | 0.3% | 0.3% | 0.3% | 0.3% | 0.3% | 0.3% | 0.3% | 0.3% |
| C Cirrhosis to Decomp | 3.0% | 3.0% | 3.0% | 3.0% | 3.0% | 3.0% | 3.0% | 3.0% | 3.0% | 3.0% | 3.0% | 3.0% | 3.0% | 3.0% | 3.0% | 3.0% | 3.0% | 3.0% |
| Low | 2.1% | 2.1% | 2.1% | 2.1% | 2.1% | 2.1% | 2.1% | 2.1% | 2.1% | 2.1% | 2.1% | 2.1% | 2.1% | 2.1% | 2.1% | 2.1% | 2.1% | 2.1% |
| High | 4.1% | 4.1% | 4.1% | 4.1% | 4.1% | 4.1% | 4.1% | 4.1% | 4.1% | 4.1% | 4.1% | 4.1% | 4.1% | 4.1% | 4.1% | 4.1% | 4.1% | 4.1% |
| C Cirrhosis to HCC | 3.6% | 3.6% | 3.6% | 3.6% | 3.6% | 3.6% | 3.6% | 3.6% | 3.6% | 3.6% | 3.6% | 3.6% | 3.6% | 3.6% | 3.6% | 3.6% | 3.6% | 3.6% |
| Low | 2.7% | 2.7% | 2.7% | 2.7% | 2.7% | 2.7% | 2.7% | 2.7% | 2.7% | 2.7% | 2.7% | 2.7% | 2.7% | 2.7% | 2.7% | 2.7% | 2.7% | 2.7% |
| High | 4.8% | 4.8% | 4.8% | 4.8% | 4.8% | 4.8% | 4.8% | 4.8% | 4.8% | 4.8% | 4.8% | 4.8% | 4.8% | 4.8% | 4.8% | 4.8% | 4.8% | 4.8% |
| Decomp to Death | 20.0% | 20.0% | 20.0% | 20.0% | 20.0% | 20.0% | 20.0% | 20.0% | 20.0% | 20.0% | 20.0% | 20.0% | 20.0% | 20.0% | 20.0% | 20.0% | 20.0% | 20.0% |
| Low | 16.0% | 16.0% | 16.0% | 16.0% | 16.0% | 16.0% | 16.0% | 16.0% | 16.0% | 16.0% | 16.0% | 16.0% | 16.0% | 16.0% | 16.0% | 16.0% | 16.0% | 16.0% |
| High | 24.0% | 24.0% | 24.0% | 24.0% | 24.0% | 24.0% | 24.0% | 24.0% | 24.0% | 24.0% | 24.0% | 24.0% | 24.0% | 24.0% | 24.0% | 24.0% | 24.0% | 24.0% |
| HCC to Death (Yr. 1) | 70.7% | 70.7% | 70.7% | 70.7% | 70.7% | 70.7% | 70.7% | 70.7% | 70.7% | 70.7% | 70.7% | 70.7% | 70.7% | 70.7% | 70.7% | 70.7% | 70.7% | 70.7% |
| Low | 43.0% | 43.0% | 43.0% | 43.0% | 43.0% | 43.0% | 43.0% | 43.0% | 43.0% | 43.0% | 43.0% | 43.0% | 43.0% | 43.0% | 43.0% | 43.0% | 43.0% | 43.0% |
| High | 77.0% | 77.0% | 77.0% | 77.0% | 77.0% | 77.0% | 77.0% | 77.0% | 77.0% | 77.0% | 77.0% | 77.0% | 77.0% | 77.0% | 77.0% | 77.0% | 77.0% | 77.0% |
| HCC to Death (Sub Yrs.) | 16.2% | 16.2% | 16.2% | 16.2% | 16.2% | 16.2% | 16.2% | 16.2% | 16.2% | 16.2% | 16.2% | 16.2% | 16.2% | 16.2% | 16.2% | 16.2% | 16.2% | 16.2% |
| Low | 11.0% | 11.0% | 11.0% | 11.0% | 11.0% | 11.0% | 11.0% | 11.0% | 11.0% | 11.0% | 11.0% | 11.0% | 11.0% | 11.0% | 11.0% | 11.0% | 11.0% | 11.0% |
| High | 23.0% | 23.0% | 23.0% | 23.0% | 23.0% | 23.0% | 23.0% | 23.0% | 23.0% | 23.0% | 23.0% | 23.0% | 23.0% | 23.0% | 23.0% | 23.0% | 23.0% | 23.0% |

| Back-Calculated Progression Rates – Females | | | | | | | | | | | | | | | | | | |
| --- | --- | --- | --- | --- | --- | --- | --- | --- | --- | --- | --- | --- | --- | --- | --- | --- | --- | --- |
| Age Cohorts | 0-  4 | 5-  9 | 10-  14 | 15-  19 | 20-  24 | 25-  29 | 30-  34 | 35-  39 | 40-  44 | 45-  49 | 50-  54 | 55-  59 | 60-  64 | 65-  69 | 70-  74 | 75-  79 | 80-  84 | 85+ |
| F0 to F1 | 4.4% | 4.4% | 4.4% | 4.4% | 4.4% | 4.4% | 4.4% | 4.4% | 11.6% | 11.6% | 14.3% | 14.3% | 16.2% | 16.2% | 18.2% | 18.2% | 18.2% | 18.2% |
| Low | 2.6% | 2.6% | 2.6% | 2.6% | 2.6% | 2.6% | 2.6% | 2.6% | 6.8% | 6.8% | 8.4% | 8.4% | 9.5% | 9.5% | 10.7% | 10.7% | 10.7% | 10.7% |
| High | 6.7% | 6.7% | 6.7% | 6.7% | 6.7% | 6.7% | 6.7% | 6.7% | 17.7% | 17.7% | 21.8% | 21.8% | 24.8% | 24.8% | 27.8% | 27.8% | 27.8% | 27.8% |
| F1 to F2 | 2.9% | 2.9% | 2.9% | 2.9% | 2.9% | 2.9% | 2.9% | 2.9% | 7.6% | 7.6% | 9.3% | 9.3% | 10.6% | 10.6% | 11.9% | 11.9% | 11.9% | 11.9% |
| Low | 1.7% | 1.7% | 1.7% | 1.7% | 1.7% | 1.7% | 1.7% | 1.7% | 4.5% | 4.5% | 5.5% | 5.5% | 6.2% | 6.2% | 7.0% | 7.0% | 7.0% | 7.0% |
| High | 4.4% | 4.4% | 4.4% | 4.4% | 4.4% | 4.4% | 4.4% | 4.4% | 11.6% | 11.6% | 14.3% | 14.3% | 16.2% | 16.2% | 18.2% | 18.2% | 18.2% | 18.2% |
| F2 to F3 | 4.5% | 4.5% | 4.5% | 4.5% | 4.5% | 4.5% | 4.5% | 4.5% | 11.9% | 11.9% | 14.6% | 14.6% | 16.6% | 16.6% | 18.6% | 18.6% | 18.6% | 18.6% |
| Low | 2.6% | 2.6% | 2.6% | 2.6% | 2.6% | 2.6% | 2.6% | 2.6% | 7.0% | 7.0% | 8.6% | 8.6% | 9.8% | 9.8% | 11.0% | 11.0% | 11.0% | 11.0% |
| High | 6.9% | 6.9% | 6.9% | 6.9% | 6.9% | 6.9% | 6.9% | 6.9% | 18.2% | 18.2% | 22.4% | 22.4% | 25.4% | 25.4% | 28.6% | 28.6% | 28.6% | 28.6% |
| F3 to C Cirrhosis | 4.7% | 4.7% | 4.7% | 4.7% | 4.7% | 4.7% | 4.7% | 4.7% | 7.7% | 7.7% | 7.7% | 7.7% | 8.7% | 8.7% | 16.7% | 16.7% | 16.7% | 16.7% |
| Low | 2.7% | 2.7% | 2.7% | 2.7% | 2.7% | 2.7% | 2.7% | 2.7% | 4.4% | 4.4% | 4.4% | 4.4% | 5.0% | 5.0% | 9.5% | 9.5% | 9.5% | 9.5% |
| High | 9.0% | 9.0% | 9.0% | 9.0% | 9.0% | 9.0% | 9.0% | 9.0% | 14.7% | 14.7% | 14.7% | 14.7% | 16.5% | 16.5% | 31.8% | 31.8% | 31.8% | 31.8% |
| F3 to HCC | 0.2% | 0.2% | 0.2% | 0.2% | 0.2% | 0.2% | 0.2% | 0.2% | 0.2% | 0.2% | 0.2% | 0.2% | 0.2% | 0.2% | 0.2% | 0.2% | 0.2% | 0.2% |
| Low | 0.2% | 0.2% | 0.2% | 0.2% | 0.2% | 0.2% | 0.2% | 0.2% | 0.2% | 0.2% | 0.2% | 0.2% | 0.2% | 0.2% | 0.2% | 0.2% | 0.2% | 0.2% |
| High | 0.3% | 0.3% | 0.3% | 0.3% | 0.3% | 0.3% | 0.3% | 0.3% | 0.3% | 0.3% | 0.3% | 0.3% | 0.3% | 0.3% | 0.3% | 0.3% | 0.3% | 0.3% |
| C Cirrhosis to Decomp | 3.0% | 3.0% | 3.0% | 3.0% | 3.0% | 3.0% | 3.0% | 3.0% | 3.0% | 3.0% | 3.0% | 3.0% | 3.0% | 3.0% | 3.0% | 3.0% | 3.0% | 3.0% |
| Low | 2.1% | 2.1% | 2.1% | 2.1% | 2.1% | 2.1% | 2.1% | 2.1% | 2.1% | 2.1% | 2.1% | 2.1% | 2.1% | 2.1% | 2.1% | 2.1% | 2.1% | 2.1% |
| High | 4.1% | 4.1% | 4.1% | 4.1% | 4.1% | 4.1% | 4.1% | 4.1% | 4.1% | 4.1% | 4.1% | 4.1% | 4.1% | 4.1% | 4.1% | 4.1% | 4.1% | 4.1% |
| C Cirrhosis to HCC | 3.6% | 3.6% | 3.6% | 3.6% | 3.6% | 3.6% | 3.6% | 3.6% | 3.6% | 3.6% | 3.6% | 3.6% | 3.6% | 3.6% | 3.6% | 3.6% | 3.6% | 3.6% |
| Low | 2.7% | 2.7% | 2.7% | 2.7% | 2.7% | 2.7% | 2.7% | 2.7% | 2.7% | 2.7% | 2.7% | 2.7% | 2.7% | 2.7% | 2.7% | 2.7% | 2.7% | 2.7% |
| High | 4.8% | 4.8% | 4.8% | 4.8% | 4.8% | 4.8% | 4.8% | 4.8% | 4.8% | 4.8% | 4.8% | 4.8% | 4.8% | 4.8% | 4.8% | 4.8% | 4.8% | 4.8% |
| Decomp to Death | 20.0% | 20.0% | 20.0% | 20.0% | 20.0% | 20.0% | 20.0% | 20.0% | 20.0% | 20.0% | 20.0% | 20.0% | 20.0% | 20.0% | 20.0% | 20.0% | 20.0% | 20.0% |
| Low | 16.0% | 16.0% | 16.0% | 16.0% | 16.0% | 16.0% | 16.0% | 16.0% | 16.0% | 16.0% | 16.0% | 16.0% | 16.0% | 16.0% | 16.0% | 16.0% | 16.0% | 16.0% |
| High | 24.0% | 24.0% | 24.0% | 24.0% | 24.0% | 24.0% | 24.0% | 24.0% | 24.0% | 24.0% | 24.0% | 24.0% | 24.0% | 24.0% | 24.0% | 24.0% | 24.0% | 24.0% |
| HCC to Death (Yr. 1) | 70.7% | 70.7% | 70.7% | 70.7% | 70.7% | 70.7% | 70.7% | 70.7% | 70.7% | 70.7% | 70.7% | 70.7% | 70.7% | 70.7% | 70.7% | 70.7% | 70.7% | 70.7% |
| Low | 43.0% | 43.0% | 43.0% | 43.0% | 43.0% | 43.0% | 43.0% | 43.0% | 43.0% | 43.0% | 43.0% | 43.0% | 43.0% | 43.0% | 43.0% | 43.0% | 43.0% | 43.0% |
| High | 77.0% | 77.0% | 77.0% | 77.0% | 77.0% | 77.0% | 77.0% | 77.0% | 77.0% | 77.0% | 77.0% | 77.0% | 77.0% | 77.0% | 77.0% | 77.0% | 77.0% | 77.0% |
| HCC to Death (Sub Yrs.) | 16.2% | 16.2% | 16.2% | 16.2% | 16.2% | 16.2% | 16.2% | 16.2% | 16.2% | 16.2% | 16.2% | 16.2% | 16.2% | 16.2% | 16.2% | 16.2% | 16.2% | 16.2% |
| Low | 11.0% | 11.0% | 11.0% | 11.0% | 11.0% | 11.0% | 11.0% | 11.0% | 11.0% | 11.0% | 11.0% | 11.0% | 11.0% | 11.0% | 11.0% | 11.0% | 11.0% | 11.0% |
| High | 23.0% | 23.0% | 23.0% | 23.0% | 23.0% | 23.0% | 23.0% | 23.0% | 23.0% | 23.0% | 23.0% | 23.0% | 23.0% | 23.0% | 23.0% | 23.0% | 23.0% | 23.0% |

**Incidence –** The following methodologies were used to estimate incidence.

The HCV model considers both horizontally and vertically acquired new infections. Vertically acquired infections were calculated by applying the mother-to-child transmission rate of HCV [5.8% (95% CI: 4.2%-7.8%)] (33) to the modeled age group-specific chronic prevalence of HCV and reported fertility rates among women of childbearing age (14). HCV-infected infants entered the disease burden model at age zero by sex, and their subsequent disease progression was tracked. The mother-to-child transmission rate of HCV is higher in HIV positive mothers [10.8% (95% CI, 7.6%-15.2%)]; however, globally coinfection is estimated to be low [2.4% (IQR 0.8-5.8%) coinfection in HIV infected individuals (34)] so coinfection was not considered for this analysis. Horizontally acquired infections were calculated separately for historical and future years.

**Historical Incidence (Horizontal) –** Historical incident cases were calibrated to reported overall, as well as sex- and age group-specific prevalence of chronic HCV infection in a country/territory using the calibration procedure described below:

*Back calculation of incidence –* When reliable prevalence estimates were available at only one point in time, a back-calculation methodology was used to estimate incidence by year. In this case, the prevalence of HCV in 1950 (who are still alive at the time of known prevalence) was assumed to be zero, and the same methodology as above was used to estimate the average annual number of new infections per year between 1950 and the year of known prevalence. The analysis was refined by developing a relative incidence curve with the 1950 relative incidence set to 1. The relative incidence was mapped based on the known risk factors and the start of blood screening. In approved models, these relative incidence curves were discussed at length with the expert panel to best estimate the historical relative number of annual incident cases of the epidemic relative to 1950. For example, in many countries the incidence of HCV was estimated to increase beginning around the 1960s or 1970s (relative to 1950), and then decrease in the 1990s as HCV screening tests became more prevalent in blood banks and transfusion centers.

The model was used to solve for a constant, times the annual relative incidence that resulted in the known prevalence after adjusting for mortality and cures. In this *calibration* step, the number of new infections shown in Equation 2 was calculated to fit the known prevalence in a given year *y* using the secant method.

Equation 2. Total HCV infections in year *y*

$\text{Total HCV Infections}_{\text{ }\mathrm{Year}_{y}\text{ }}\text{=}\sum_{\text{t}\text{=1950}}^{\text{ }\text{y}} \left( \text{New Infections}_{\text{t}} \text{– }\text{Spontaneously Cured}_{\text{t}}\text{– }\text{Mortality}_{\text{t}} \text{–}\text{ }\text{Cured}_{\text{t}} \right)$

The annual incidence cases were distributed by age and sex, and the modeled distribution was compared to the reported distribution. An iterative process of modifying the relative incidence curve and allocation by age was used to match the two curves and estimate the annual number of new infections by year.

**Future Incidence (Horizontal) –** Future incident cases were calculated in the model and were assumed to change at the same annual rate as chronic prevalent cases (F0 or overall) of HCV infection, relative to the last year of incidence data. The rate of change in incident cases was based on the rate of change in all chronic prevalent cases.

**Screening module** **methodology–** A screening module was added to calculate the number of screens necessary for diagnosing a given number of HCV-infected cases. The screening module used the inputs and assumptions of the model (annual population by age, number of annual new diagnoses) in conjunction with the outputs (undiagnosed HCV-infected population that was either asymptomatic or not linked-to-care) to calculate the number of annual HCV antibody and HCV RNA tests necessary for a given scenario. Only cases that were either asymptomatic or not yet linked to care were considered as part of the case-finding algorithm. It was assumed that cases with advanced liver disease were symptomatic and would seek care with or without a screening strategy. Therefore, each newly diagnosed case of advanced liver disease would require one HCV antibody and one HCV RNA test, regardless of the screening strategy.

Since the share of symptomatic and linked-to-care cases varies by stage of liver disease, the input number of newly diagnosed cases was segmented by stage of liver disease, with the more advanced stages being diagnosed first. The modeled disease burden was used in this segmentation. It was also assumed that cases of spontaneously cleared HCV infection were diagnosed at the same rate as the asymptomatic chronically infected cases.

To calculate the size of the population eligible for screening, the module tracked the populations with a history of screening, diagnosis, or SVR. The number of HCV antibody screens each person could receive (1, 2 or unlimited) was specified based on country screening policies as follows. The default assumption was that in Belgium individuals would be screened (on average) a maximum of two times, without a robust national screening program. Finally, a prevalence multiplier was considered for countries/territories where screening programs were limited to limited to persons with a risk factor for HCV. The default multiplier was set to 5, assuming that the population tested for HCV (in the absence of a robust national screening program) had a prevalence that was 5-times higher than the general population in the country/territory.

The number needed to screen (NNS) to find one HCV antibody-positive case was calculated as shown in Equations 3–4. Next, the number of annual HCV antibody screens performed was calculated as shown in Equation 5. Lastly, every newly diagnosed HCV antibody-positive case was assumed to be followed up with an HCV RNA test.

**Equation 3.** Number needed to screen to diagnose one HCV antibody-positive case, unadjusted, in year $t$

$\text{NNS}_{t}^{*}=\frac{1}{\frac{\text{Undiagnosed, asymptomatic or not linked-to-care HCV antibody-positive cases}_{t}}{\text{Population eligible for screening}_{t}}}$, where

$\text{Population eligible for screening}_{t}$was the population eligible for screening in year $t$. Eligibility of screening was determined by birth year (by scenario), history of having received a screen within the screening strategy (excluded), history of diagnosis (excluded), and history of SVR (excluded).

**Equation 4.** Number needed to screen to diagnose one HCV antibody-positive case, adjusted, in year $t$

$\text{NNS}_{t}=\frac{\text{NNS}_{t}^{*}}{k}$, where

$\text{NNS}_{t}^{*}$is the number needed to screen to diagnose one HCV antibody-positive case, unadjusted, in year $t$;

$k$ is the ratio of prevalence in the population where the screening is conducted to that in the general population.

**Equation 5.** Number of HCV antibody screens performed, in year $t$

$\text{Newly diagnosed symptomatic HCV antibody-positive cases}_{t}+\text{NNS}_{t}\times\text{Newly diagnosed asymptomatic or not yet linked-to-care cases}_{t}$, where

$\text{NNS}_{t}$is the number needed to screen to diagnose one HCV antibody-positive case in year $t$.

**Validation of the model –** The model was validated by comparing its output against empirical data. In the US (35, 36), France (37, 38) and Egypt (39, 40), at least two robust prevalence studies were available for comparison. In addition, the incidence of HCC cases was available through Globocan (41). Studies that reported a percent of all HCC cases due to HCV (12, 42-57) were used to adjust the reported HCC cases and compare them against the model output. Additionally, the model outcomes were reviewed with the expert panel and compared against expectations for future changes in prevalence, incidence, and end stage outcomes.

Figure 1. Reported and modeled prevalent viremic HCV cases – France, 2004-2020

# References

1. Razavi H, Estes C, Pasini K, Gower E, Hindman S. HCV treatment rate in select European countries in 2004-2010. Journal of Hepatology. 2013;58:S22-S3.

2. Razavi H, Waked I, Sarrazin C, Myers RP, Idilman R, Calinas F, et al. The present and future disease burden of hepatitis C virus (HCV) infection with today's treatment paradigm. J Viral Hepat. 2014;21 Suppl 1:34-59.

3. Blach S, Zeuzem S, Manns M, Altraif I, Duberg A-S, Muljono DH, et al. Global prevalence and genotype distribution of hepatitis C virus infection in 2015: a modelling study. The Lancet Gastroenterology & Hepatology. 2016;2(3):161-76.

4. Litzroth A, Suin V, Wyndham-Thomas C, Quoilin S, Muyldermans G, Vanwolleghem T, et al. Low hepatitis C prevalence in Belgium: implications for treatment reimbursement and scale up. BMC Public Health. 2019;19(1):39.

5. De Maeght S, Henrion J, Bourgeois N, de Galocsy C, Langlet P, Michielsen P, et al. A pilot observational survey of hepatitis C in Belgium. Acta Gastroenterol Belg. 2008;71(1):4-8.

6. Gerkens S, Martin N, Thiry N, Hulstaert F. [Hepatitis C: Screening and Prevention] HEPATITIS C: SCREENING EN PREVENTIE. 2012 2012.

7. Cejas NG, Villamil FG, Lendoire JC, Tagliafichi V, Lopez A, Krogh DH, et al. Improved waiting-list outcomes in Argentina after the adoption of a model for end-stage liver disease-based liver allocation policy. Liver Transpl. 2013;19(7):711-20.

8. Šperl J, Fraňková S, Trunečka P. [Liver transplantation for chronic hepatitis C, the importance of antiviral treatment]. Gastroent Hepatol. 2013;67(5):407-12.

9. Carmiel-Haggai M. [Two decades of liver transplantation in Israel]. Harefuah. 2012;151(12):679-83, 721.

10. Krawczyk M, Grat M, Barski K, Ligocka J, Antczak A, Kornasiewicz O, et al. 1000 liver transplantations at the Department of General, Transplant and Liver Surgery, Medical University of Warsaw--analysis of indications and results. Pol Przegl Chir. 2012;84(6):304-12.

11. Adam R, Karam V, Delvart V, O'Grady J, Mirza D, Klempnauer J, et al. Evolution of indications and results of liver transplantation in Europe. A report from the European Liver Transplant Registry (ELTR). J Hepatol. 2012;57(3):675-88.

12. Yang JD, Kim B, Sanderson SO, St Sauver JL, Yawn BP, Pedersen RA, et al. Hepatocellular carcinoma in olmsted county, Minnesota, 1976-2008. Mayo Clin Proc. 2012;87(1):9-16.

13. World Population Prospects: The 2017 Revision [Internet]. 2017. Available from: <https://esa.un.org/unpd/wpp>.

14. World Population Prospects 2019, Online Edition [Internet]. United Nations, Department of Economic and Social Affairs, Population Division. 2019 [cited 2019-09-20]. Available from: <https://population.un.org/wpp/>.

15. Engstrom A, Adamsson C, Allebeck P, Rydberg U. Mortality in patients with substance abuse: A follow-up in Stockholm County, 1973-1984. Int J Addict. 1991;26(1):91-106.

16. Frischer M, Goldberg D, Rahman M, Berney L. Mortality and survival among a cohort of drug injectors in Glasgow, 1982-1994. Addiction. 1997;92(4):419-27.

17. Hickman M, Carnwath Z, Madden P, Farrell M, Rooney C, Ashcroft R, et al. Drug-related mortality and fatal overdose risk: Pilot cohort study of heroin users recruited from specialist drug treatment sites in London. J Urban Health. 2003;80(2):274-87.

18. Oppenheimer E, Tobutt C, Taylor C, Andrew T. Death and survival in a cohort of heroin addicts from London clinics: A 22-year follow-up study. Addiction. 1994;89(10):1299-308.

19. Perucci CA, Davoli M, Rapiti E, Abeni DD, Forastiere F. Mortality of intravenous drug users in Rome: A cohort study. Am J Public Health. 1991;81(10):1307-10.

20. Bjornaas MA, Bekken AS, Ojlert A, Haldorsen T, Jacobsen D, Rostrup M, et al. A 20-year prospective study of mortality and causes of death among hospitalized opioid addicts in Oslo. BMC Psychiatry. 2008;8:8.

21. Kamper-Jorgensen M, Ahlgren M, Rostgaard K, Melbye M, Edgren G, Nyren O, et al. Survival after blood transfusion. Transfusion. 2008;48(12):2577-84.

22. Aceijas C, Stimson GV, Hickman M, Rhodes T. Global overview of injecting drug use and HIV infection among injecting drug users. AIDS. 2004;18(17):2295-303.

23. Nelson PK, Mathers BM, Cowie B, Hagan H, Des Jarlais D, Horyniak D, et al. Global epidemiology of hepatitis B and hepatitis C in people who inject drugs: Results of systematic reviews. The Lancet. 2011;378(9791):571-83.

24. UNAIDS. THE GAP REPORT 2014: People who inject drugs. Online: Joint United Nations Programme on HIV/AIDS; 2014.

25. Razavi H, Elkhoury AC, Elbasha E, Estes C, Pasini K, Poynard T, et al. Chronic hepatitis C virus (HCV) disease burden and cost in the United States. Hepatology. 2013;57(6):2164-70.

26. Harris RJ, Thomas B, Griffiths J, Costella A, Chapman R, Ramsay M, et al. Increased uptake and new therapies are needed to avert rising hepatitis C-related end stage liver disease in England: modelling the predicted impact of treatment under different scenarios. J Hepatol. 2014;61(3):530-7.

27. Thein HH, Yi Q, Dore GJ, Krahn MD. Natural history of hepatitis C virus infection in HIV-infected individuals and the impact of HIV in the era of highly active antiretroviral therapy: a meta-analysis. AIDS. 2008;22(15):1979-91.

28. Surveillance, Epidemiology, and End Results (SEER) Program Research Data (1973-2013) [Internet]. National Cancer Institute. 2016 [cited August 10th 2016]. Available from: [www.seer.cancer.gov](file://vmware-host/Shared%20Folders/Dropbox%20(CfDA)/CDA%20Library/Manuscripts/2022%20S%20(Busschots_Ho)%20Belgium%20HCV%20Update/BMC%20Resubmission/www.seer.cancer.gov).

29. Altekruse SF, Henley SJ, Cucinelli JE, McGlynn KA. Changing hepatocellular carcinoma incidence and liver cancer mortality rates in the United States. Am J Gastroenterol. 2014;109(4):542-53.

30. Bernfort L, Sennfalt K, Reichard O. Cost-effectiveness of peginterferon alfa-2b in combination with ribavirin as initial treatment for chronic hepatitis C in Sweden. Scand J Infect Dis. 2006;38(6-7):497-505.

31. Ries L, Young G, Keel G, Eisner M, Lin Y, Horner M. SEER survival monograph: Cancer survival among adults: U.S. SEER program, 1988-2001, patient and tumor characteristics. Bethesda, MD: National Cancer Institute, SEER Program; 2007.

32. Harris RJ, Thomas B, Griffiths J, Costella A, Chapman R, Ramsay M, et al. Increased uptake and new therapies are needed to avert rising hepatitis C-related end stage liver disease in England: Modelling the predicted impact of treatment under different scenarios. J Hepatol. 2014;61(3):530-7.

33. Benova L, Mohamoud YA, Calvert C, Abu-Raddad LJ. Vertical transmission of hepatitis C virus: systematic review and meta-analysis. Clin Infect Dis. 2014;59(6):765-73.

34. Platt L, Easterbrook P, Gower E, McDonald B, Sabin K, McGowan C, et al. Prevalence and burden of HCV co-infection in people living with HIV: a global systematic review and meta-analysis. Lancet Infect Dis. 2016;16(7):797-808.

35. Armstrong GL, Wasley A, Simard EP, McQuillan GM, Kuhnert WL, Alter MJ. The prevalence of hepatitis C virus infection in the United States, 1999 through 2002. Ann Intern Med. 2006;144(10):705-14.

36. Denniston MM, Jiles RB, Drobeniuc J, Klevens RM, Ward JW, McQuillan GM, et al. Chronic hepatitis C virus infection in the United States, National Health and Nutrition Examination Survey 2003 to 2010. Ann Intern Med. 2014;160(5):293-300.

37. Meffre C, Le Strat Y, Delarocque-Astagneau E, Dubois F, Antona D, Lemasson JM, et al. Prevalence of hepatitis B and hepatitis C virus infections in France in 2004: social factors are important predictors after adjusting for known risk factors. J Med Virol. 2010;82.

38. Brouard C, Pillonel J, Boussac M, de Lédinghen V, Rachas A, Silvain C, et al. French hepatitis C care cascade: substantial impact of direct-acting antivirals, but the road to elimination is still long. BMC Infect Dis. 2020;20(1):759.

39. El-Zanaty F, Way A. Egypt demographic and health survey, 2008. Cairo: Cairo, Egypt : Ministry of Health and Population, 2009; 2009. p. 431.

40. Ministry of Health and Population [Egypt], El-Zanaty and Associates [Egypt], ICF International. Egypt Health Issues Survey. Cairo, Egypt; 2015.

41. GLOBOCAN. GLOBOCAN 2012: Estimated Cancer Incidence, Mortality and Prevalence Worldwide in 2012 2012 [Available from: <http://globocan.iarc.fr/Default.aspx>.

42. El-Serag HB. Epidemiology of viral hepatitis and hepatocellular carcinoma. Gastroenterology. 2012;142(6):1264-73.

43. Dondog B, Lise M, Dondov O, Baldandorj B, Franceschi S. Hepatitis B and C virus infections in hepatocellular carcinoma and cirrhosis in Mongolia. European Journal of Cancer Prevention. 2011;20(1):33-9.

44. Raza SA, Clifford GM, Franceschi S. Worldwide variation in the relative importance of hepatitis B and hepatitis C viruses in hepatocellular carcinoma: A systematic review. British Journal of Cancer. 2007;96(7):1127-34.

45. Stroffolini T, Trevisani F, Pinzello G, Brunello F, Tommasini MA, Iavarone M, et al. Changing aetiological factors of hepatocellular carcinoma and their potential impact on the effectiveness of surveillance. Digestive and Liver Disease. 2011;43(11):875-80.

46. Weinmann A, Koch S, Niederle IM, Schulze-Bergkamen H, Konig J, Hoppe-Lotichius M, et al. Trends in epidemiology, treatment, and survival of hepatocellular carcinoma patients between 1998 and 2009: An analysis of 1066 cases of a German HCC registry. J Clin Gastroenterol. 2013.

47. Deuffic-Burban S, Deltenre P, Buti M, Stroffolini T, Parkes J, Muhlberger N, et al. Predicted effects of treatment for HCV infection vary among European countries. Gastroenterology. 2012;143(4):974-85.e14.

48. Harnois DM. Hepatitis C Virus Infection and the Rising Incidence of Hepatocellular Carcinoma. Mayo Clin Proc. 2012;87(1):7-8.

49. Fares N, Peron JM. [Epidemiology, natural history, and risk factors of hepatocellular carcinoma]. La Revue du praticien. 2013;63(2):216-7, 20-2.

50. Hezode C, editor The changing pattern of epidemiology in hepatocellular carcinoma.

51. Hong TP, Gow P, Fink M, Dev A, Roberts S, Nicoll A, et al. Novel population-based study finding higher than reported hepatocellular carcinoma incidence suggests an updated approach is needed. Hepatology. 2016;63(4):1205-12.

52. Pocobelli G, Cook LS, Brant R, Lee SS. Hepatocelluar carcinoma incidence trends in Canada: analysis by birth cohort and period of diagnosis. Liver International. 2007:1272-9.

53. Sanyal A, Poklepovic A, Moyneur E, Barghout V. Population-based risk factors and resource utilization for HCC: US perspective. Current Medical Research and Opinion. 2010;26(9):2183-91.

54. Shibuya K, Yano E. Regression analysis of trends in mortality from hepatocellular carcinoma in Japan, 1972-2001. International Journal of Epidemiology. 2005;34:397-402.

55. Stroffolini T. Etiological factor of hepatocellular carcinoma in Italy. Minerva Gastroenterol Dietol. 2005;51(1):1-5.

56. Tokushige K, Hashimoto E, Yatsuji S, Tobari M, Taniai M, Torii N, et al. Prospective study of hepatocellular carcinoma in nonalcoholic steatohepatitis in comparison with hepatocellular carcinoma caused by chronic hepatitis C. J Gastroenterol. 2010;45:960-7.

57. (OPTN) OPaTN. National Data. 2013 2013.
