# Supplementary material for: Ten years countdown to hepatitis C elimination in Belgium: a mathematical modeling approach
Source: BMC Infect Dis. 2022 Apr 22;22:397. doi: 10.1186/s12879-022-07378-3 (PMC9026052; doi:10.1186/s12879-022-07378-3)
Supplement: Supplementary file 2 — Additional file 2: Fig. S1. Sensitivity analysis and 95% uncertainty intervals of Belgian viremic HCV prevalence. [file 12879_2022_7378_MOESM2_ESM.docx]

**Additional File 2**

**Figure S1. Sensitivity analyses**

Note: Top 10 drivers of uncertainty, arranged from most uncertainty (top) to least uncertainty (bottom). Numbers next to the bars represent the

input range (i.e. if Belgium’s prevalence was 46,600 in 2015 then the 2020 prevalence would be 43,000)

**Table S1. 95% uncertainty intervals (Beginning of year viremic prevalence, rounded)**

|  | **2015** | **2016** | **2017** | **2018** | **2019** | **2020** |
| --- | --- | --- | --- | --- | --- | --- |
| Base | 20,000 | 18,800 | 18,100 | 16,400 | 15,500 | 13,100 |
| Low | 9,300 | 8,500 | 8,000 | 7,100 | 6,500 | 4,200 |
| High | 43,000 | 41,600 | 40,700 | 38,800 | 37,800 | 35,300 |
